# Prenatal Exposure to a Climate-Related Disaster Results in Changes of the Placental Transcriptome and Infant Temperament

**DOI:** 10.3389/fgene.2022.887619

**Published:** 2022-04-29

**Authors:** Jessica Buthmann, Dennis Huang, Patrizia Casaccia, Sarah O’Neill, Yoko Nomura, Jia Liu

**Affiliations:** ^1^ Department of Psychology, Stanford University, Stanford, CA, United States; ^2^ The Graduate Center at the City University of New York, New York, NY, United States; ^3^ Advanced Science Research Center at the Graduate Center, Neuroscience Initiative, City University of New York, New York, NY, United States; ^4^ The City College of New York at the City University of New York, New York, NY, United States; ^5^ Department of Psychology, Queens College, City University of New York, New York, NY, United States

**Keywords:** Superstorm Sandy, surgency/extraversion, placenta, inflammation, sensory perception, epigenome

## Abstract

Maternal stress during pregnancy exerts long-term effects on the mental well-being of the offspring. However, the long-term effect of prenatal exposure on the offspring’s mental status is only partially understood. The placenta plays a vital role in connecting the maternal side to the fetus, thereby serving as an important interface between maternal exposure and fetal development. Here, we profiled the placental transcriptome of women who were pregnant during a hurricane (Superstorm Sandy), which struck New York City in 2012. The offspring were followed longitudinally and their temperament was assessed during the first 6–12 months of age. The data identified a significant correlation between a Superstorm Sandy stress factor score and infant temperament. Further, analysis of the placental transcriptomes identified an enrichment of functional pathways related to inflammation, extracellular matrix integrity and sensory perception in the specimen from those infants with “Slow-to-Warm-up” temperament during the first year of life. Together, these findings provide initial evidence that maternal exposure to climate-related disasters results in altered placental transcriptome, which may be related to long-term emotional and behavioral consequences in children.

## Introduction

The prenatal period is a particularly vulnerable time for disruption because of the rapid pace of development. The Developmental Origins of Health and Disease (DOHaD) hypothesis, first posited by Barker (Barker, 1998), states that gestational conditions can affect the development of offspring with health consequences in both the short and long term. Having first made this connection in the context of birth weight and adult health and illness, researchers across disciplines have studied the underlying causes of associations between early conditions and subsequent wellbeing. As such, the DOHaD hypothesis (Gluckman et al., 2007) has provided a framework for investigations into the effects of a spectrum of early life factors, both pre- and postnatal, on an organism’s wellbeing and the underlying biological mechanisms.

A large body of literature provides evidence for this hypothesis in the context of prenatal stress and suboptimal offspring emotional, behavioral and cognitive development. Prenatal maternal stress (PNMS) related to normative stressors (Zhu et al., 2014), psychopathology (Davis et al., 2007), and stressful events such as natural disasters (Buthmann et al., 2019) has been shown to predict worse outcomes in children, including temperament and anxiety symptoms. Researchers have posited that the maternal physiological stress response, including inflammation and cortisol increase, signals the presence of stressful environment conditions to the developing fetus, leading to a cascade of adaptations to better prepare the offspring for those conditions (Weinstock, 2008). However, the precise mechanisms underlying the effect of PNMS on offspring’s neurobehavioral outcomes remain not fully understood.

Serving at the maternal-fetal interface, the placenta has been shown to play a critical role in transferring nutrients and oxygen, regulating the secretion of hormones and growth factors, and protecting the fetus from maternal experiences during pregnancy, including maternal stress. Maternal exposure to storm stress was previously linked with offspring anxiety, aggression, and higher levels of hair cortisol and dehydroepiandrosterone detected at 3–4 years of age (Nomura et al., 2021). Besides hormonal changes, altered placental gene expression has also been reported in response to maternal exposure to natural disasters, such as Superstorm Sandy, and has been correlated with offspring temperament. For example, placental levels of *MAOA (Monoamine Oxidase A)* transcripts were found to partially mediate the association between prenatal storm exposure and the amount of smiling and laughter exhibited by offspring at 2 months of age (Pehme et al., 2018). Higher levels of placental *HSD11B1* (*Hydroxysteroid 11-Beta Dehydrogenase 1*) transcripts were also found to predict lower Negative Affectivity, while higher levels of *NCOR2 (Nuclear Receptor Corepressor 2)* transcripts were found to be predictive of higher distress to limitations (i.e., greater frustration) at 2 years of age (Finik et al., 2020). How these transcriptional alterations in the placenta may link to behavioral alteration in the offspring is not fully understood; however, these data suggest that studying the placental transcriptomic provides important information which help in predicting early infant temperament and neurodevelopment. Such knowledge is of great importance to allow the development and implementation of strategies for early life intervention, prior to the onset of clinical symptoms in childhood.

The goal of this study was to identify the placental transcriptomic changes induced by maternal exposure to climate-related disasters, and define their potential association with early child temperament profiles in the offspring. For this reason, we assessed the temperament profiles of children whose mothers experienced Superstorm Sandy, a destructive hurricane that affected the New York metropolitan area in October of 2012. Using RNA-seq, we identified differential placental gene expression profiles in children from mothers with hurricane exposure, and discuss potential pathways identified in the placenta specimens that were related to distinct temperament profiles.

## Materials and Methods

**Study Population.** Pregnant women were recruited at their prenatal clinic in the New York City area into a longitudinal birth cohort study. Exclusion criteria included HIV infection, maternal psychosis, maternal age less than 15 years, and life-threatening medical complications. All participants provided written informed consent and agreed to be longitudinally followed-up. Institutional review board approval was obtained from Mt. Sinai Hospital, New York Presbyterian Queens Hospital, and the City University of New York Queens College. The recruitment period included the time when Superstorm Sandy affected the New York area. Detailed information on the study can be found in the study’s cohort profile (Finik and Nomura, 2017). A subsample of participants from whom placenta tissue was collected at delivery and completed self-report questionnaires were available and are included in the current study (*n* = 33). Among all participants, all mothers were exposed to Superstorm Sandy during pregnancy: 9.1% during the 3rd trimester, 27.3% during 2nd trimester, and 63.6% during the 1st trimester. The description and demographics of the included participants is provided in Table 1.

**TABLE 1:** Exposure and demographic characteristics of the full sample and by temperament group.

	Full sample (N = 33)	Easy temperament (N = 11)	Intermediate temperament (N = 11)	Slow-to-Warm-up temperament (N = 11)
Ethnicity (% Hispanic)	63.6%	54.5%	81.2%	63.6%
Marital status (% married)	69.7%	72.3%	72.3%	36.4%
Educational attainment (% College+)	48.5%	45.5%	72.3%	27.3%
Financial assistance	15.0%	27.3%	0.0%	18.2%
Exposure timing in gestation (weeks) Mean (SD)	10.28 (11.14)	10.10 (11.26)	9.97 (10.17)	10.75 (12.90)
Child sex (% female)	51.2%	45.5%	63.6%	45.5%
Surgency/Extraversion M(SD)	5.4 (0.65)	5.8 (0.57)	5.5 (0.40)	4.8 (0.58)
Negative Affectivity M(SD)	3.5 (0.76)	3.4 (0.96)	3.4 (0.69)	3.8 (0.56)
Orienting/Regulation M(SD)	5.2 (0.62)	5.4 (0.78)	5.3 (0.38)	4.9 (0.56)

**Tissue collection and RNA extraction.** Placentas were sampled as previously described (Finik and Nomura, 2017; Nomura et al., 2021). Briefly, placenta biopsies, free of maternal decidua, were excised from each placenta quadrant, snap frozen in liquid nitrogen for 24 h and then stored at −80°C. RNA extraction was carried out by first grinding frozen tissue in a liquid nitrogen-cooled mortar. RNA was extracted with the Maxwell 16 automated DNA/RNA extraction equipment (Promega: Madison, WI) using the proprietary extraction kits following the manufacturer’s protocol. RNA was quantified with Nanodrop spectrophotometer (Thermo Electron North America: Madison, WI) and stored at −80°C.

**Temperament Measures**. Mothers reported on their children’s temperament at 6–12 months postpartum using the Infant Behavior Questionnaire Revised (IBQ-R; (Gartstein and Rothbart, 2003); on paper. This 91-item measure assesses the frequency with which the child engages in specific behaviors reflective of temperament characteristics within the past week. Respondents rate the frequency of behavior on a scale from 1 (never) to 7 (always). The items in the measure form 14 subscales, which are combined into 3 higher-order temperament dimensions. The Negative Affectivity dimension comprises sadness, distress to limitations, fear, and reverse-coded falling reactivity subscales. Similarly, Orienting/Regulation consists of four subscales, including cuddliness duration of orientating, soothability, and pleasure from low intensity stimuli subscales. Lastly, Surgency/Extraversion consists of six subscales, including activity level, smiling and laughter, approach, perceptual sensitivity, vocal reactivity, and pleasure from high intensity stimuli subscales. This measure has been shown to have adequate discriminate and convergent validity, internal consistency, and interrater reliability between primary and secondary caregivers in populations 3–12 months of age (Gartstein and Rothbart, 2003; Parade and Leerkes, 2008). Gartstein and Rothbart recommended using intra-sample values to determine high and low scores, rather than norms developed in different samples. As described below, we created a factor score based on Negative Affectivity, Orienting/Regulation, and Surgency/Extraversion, and refer to high, moderate, and low scores as “Easy,” “Intermediate” and “Slow-to-Warm-up” temperament profiles, respectively.

**Superstorm Sandy Stress**. Subjective experience of the storm was measured using the Posttraumatic Diagnostic Scale or PDS (Foa, 1995) reported by mothers. This 49-item self-report measure assesses the severity of posttraumatic stress disorder (PTSD) symptoms due to Superstorm Sandy. The participant reports the frequency with which they experienced each of the 49 listed symptoms on a 4-point scale from 0 “not at all or only one time” to 3 “5 or more times a week/almost always.” This was administered shortly after the storm occurred, or at the time of enrollment into the study (M = 0.79, SD = 1.80).

Objective experience of the storm was reported by participants using Storm32, developed by King & Laplante (King and Laplante, 2005). The measure yields four subscales: Threat, which reflects actual or threat of injury to the participant or their loved ones due to the storm (M = 0.48, SD = 0.91); Loss, which reflects financial loss due to the storm (M = 0.97, SD = 1.74); change, which reflects disrupted living arrangements due to the storm (M = 0.94, SD = 0.79); and Scope, which reflects time without access to phone or electricity due to the storm (M = 0.18, SD = 0.70). There is no missing data in this study.

**Maternal Psychopathology**. Assessments were done between 24 and 28 weeks of gestation. A Global Assessment of Functioning (GAF) was assessed (M = 86.52, SD = 6.19) by the Structured Clinical Interview for DSM-IV (SCID-IV). GAF is single score representing global functioning. It is generated by integrating information about a person’s level of impairment/functioning and symptom severity. Lower scores are indicative of more severe pathology/lower functioning (APA, 2000). Prenatal maternal depression was measured by the Edinburgh Postnatal Depression Scale (EPDS) (Murray and Carothers, 1990). Participants reported how often they experienced ten of the listed symptoms of depression in the past week on a four-point scale from “yes, all of the time,” to “no, not at all.” Mean (SD) of the depressive symptom was 8.03 (3.72) and Cronbach’s alpha was 0.801. State and trait anxiety were measured using the State-Trait Anxiety Inventory (STAI) (Spielberger, 1983). State and Trait anxiety were each assessed by 20 items, with participants rating how well each item described them on a four-point scale from “not at all” to “very much so.” Mean (SD) for State and Trait anxiety symptoms were 36.23 (9.63) and 35.81 (10.23) respectively. Cronbach’s alpha for State and Trait anxiety was 0.896 and 0.854 respectively.

**Socioeconomic status (SES).** Participants reported whether they experienced financial difficulty and/or received financial public assistance during pregnancy. No financial difficulty was coded 2, some financial difficulty was coded as 1, and having received financial public assistance was coded as 0. Participants self-reported their level of educational attainment. Having completed a graduate degree was coded as 3, an associate’s or bachelor’s degree was coded as 2, a high school degree or equivalent was coded as a 1, and less than a high school degree was coded as a 0. Education and financial difficulty were summed, with a higher score reflecting higher SES.

## Statistical Analysis

**Temperament profile and maternal mental health.** The three temperament composite scores, Surgency/Extraversion, Negative Affectivity, and Orienting/Regulation, were standardized and transformed into z-scores to improve normal distribution. All three composite scores were weighted by 0.1 times maternal socioeconomic status (SES) and presence of a substance dependence. The weighted z-scores of the three composite scores were entered into a principal components analysis with varimax rotation. The Kaiser-Meyer-Olkin measure of sampling adequacy was 0.59 and Bartlett’s test of sphericity was significant (X^2^(3) = 14.28, *p* = 0.003). One factor emerged, explaining 57.47% of the variance. Scores were rank ordered and participants with the top and bottom tertile scores were selected for transcriptome analyses to compare the most extreme cases (Table 2).

**TABLE 2:** Correlations among variables of interest.

	1	2	3	4	5	6	7	8	9	10	11	12	13	14	15
1. Surgency/Extraversion	–	–	–	–	–	–	–	–	–	–	–	–	–	–	–
2. Negative Affectivity	−0.09	–	–	–	–	–	–	–	–	–	–	–	–	–	–
3. Orienting/Regulation	0 0.44*	−0.51**	–	–	–	–	–	–	–	–	–	–	–	–	–
4. IBQ Factor Score	0.62***	−0.34^†^	0.42*	–	–	–	–	–	–	–	–	–	–	–	–
5. Storm PTSD Symptoms	−0.22	0.28	−0.39*	−0.10	–		–	–	–	–	–	–	–	–	–
6. Storm32 Threat	−0.21	0.29^†^	−0.06	−0.30^†^	0.45**	–	–	–	–	–	–	–	–	–	–
7. Storm32 Loss	−0.35*	0.31^†^	−0.09	−0.26	0.63***	0.60***	–	–	–	–	–	–	–	–	–
8. Storm32 Scope	−0.18	0.12	−0.22	−0.31^†^	0.72***	0.28	0.68***	–	–	–	–	–	–	–	–
9. Storm32 Change	0.01	0.31^†^	−0.08	−0.11	0.30^†^	0.17	0.36*	0.26	–	–	–	–	–	–	–
10. Storm Factor	−0.31^†^	0.33^†^	−0.25	−0.32^†^	0.87***	0.65***	0.90***	0.83***	0.52**	–	–	–	–	–	–
11. Global Assessment of Functioning	−0.01	−0.21	−0.10	0.00	0.17	0.09	−0.03	0.12	0.02	−0.03	–		–	–	–
12. Prenatal Depression	−0.01	0.32^†^	−0.05	0.26	0.06	−0.04	0.04	−0.26	0.17	−0.03	0.36*	–	–	–	–
13. State Anxiety	0.12	0.15	−0.12	0.34^†^	0.18	−0.05	−0.03	−0.15	−0.03	−0.01	0.41*	0.50**	–	–	–
14. Trait Anxiety	0.15	0.28	−0.11	0.29	0.15	−0.11	−0.03	−0.15	−0.07	−0.04	−0.40*	0.59***	0.82***	–	–
15. Psychopathology Factor	0.08	0.29	−0.06	0.30^†^	0.21	−0.09	0.01	−0.21	0.04	0.00	−0.64**	0.77***	0.87***	0.90***	–

^†^ *p* < 0.1, ∗*p* < 0.05, ∗∗*p* < 0.01, ∗∗∗*p* < 0.001. Numbers across the top row correspond to the variables in the first column. Matrix values represent Pearson’s correlation coefficients.

Prenatal depression score, trait anxiety score, state anxiety score, GAF, PTSD symptoms related to Superstorm Sandy, and Storm32 Change, Loss, Scope, and Threat were standardized by z-score transformation and entered into a principal components analysis with varimax rotation. The Kaiser-Meyer-Olkin measure of sampling adequacy was 0.60 and Bartlett’s test of sphericity was significant (X^2^(36) = 126.71, *p* < 0.001). Two factors emerged, explaining 61.98% of the variance in total. The first factor comprised the Superstorm Sandy-related stress measures. The second comprised the maternal psychopathology measures. All rotated factor loadings were greater than or equal to 0.52.

**Hierarchical linear regression.** In step one, demographic confounders, including marital status, race, and SES were entered. Next, the maternal psychopathology factor was entered in step two, and storm stress factor in step three to assess the unique contribution of maternal psychopathology and storm stress in explaining the variance in infant temperament.

**Transcriptome analysis.** RNA-seq was performed at Novagene Corp. (Sacramento, CA). Ribosomal RNA was removed using Ribo-Zero Kit. mRNA was fragmented randomly by adding fragmentation buffer, then the cDNA was synthesized using mRNA template and random hexamer primers, after which a custom second-strand synthesis buffer (Illumina), dNTPs, RNase H and DNA polymerase I were added to initiate the second-strand synthesis. After a series of terminal repair, ligation and sequencing adaptor ligation, the double-stranded cDNA library was completed through size selection and PCR enrichment. Bulk RNA sequencing data was processed with the trimmomatic tool (Bolger et al., 2014) and mapped to the hg38 reference genome by the subread aligner (Liao et al., 2013). Mapped reads were summarized with the featureCounts tool of the subread package against Ensembl’s human gene annotation (ENSEMBL.GRCh38. v98). Entire dataset was deposited into GEO data depositary (accession number GSE197697). Differential expression analysis was performed by DESeq2 (Love et al., 2014) in R, after low count genes were removed by filtering out genes with total raw counts of less than 10 summed across all samples. Only protein-coding genes were used for ontology construction. Based on filtering criteria (base mean > 5, *p* < 0.05 and fold change >1.5), we identified a total of 646 differentially expressed genes (Supplementary Table S1). The list of differentially expressed genes was also used to perform *Enrichr* analysis (Chen et al., 2013; Kuleshov et al., 2016; Xie et al., 2021). We further used the genes pre-ranked by fold change to perform a GSEA pre-ranked analysis using the curated gene datasets C2 and C5 (Mootha et al., 2003; Subramanian et al., 2005).

## Results

**FIGURE 1:**
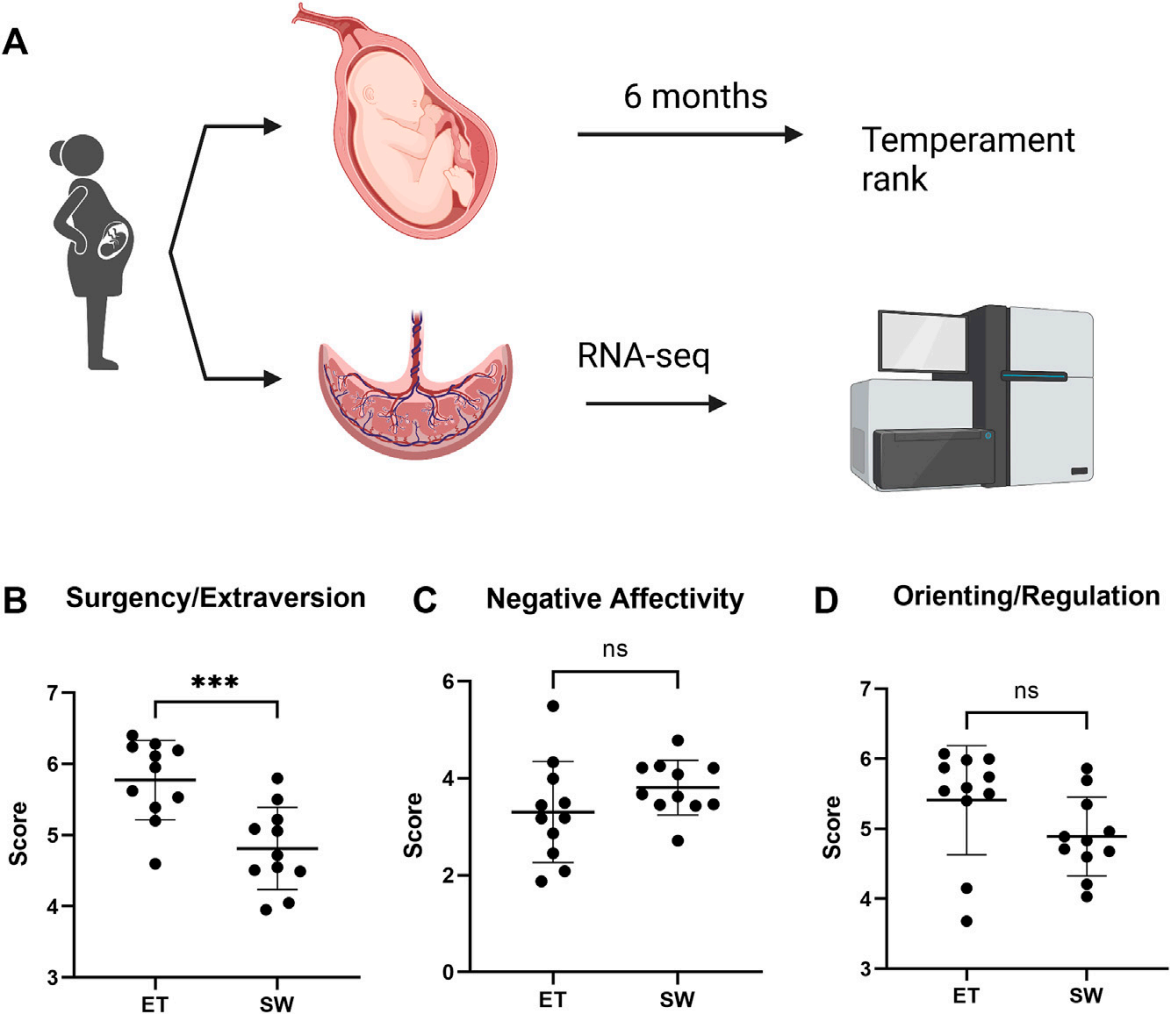
Assessment of infant temperament score at 6 months of age in children exposed to Superstorm Sandy *in utero*. **(A)** Experiment outline **(B)** Temperament was assessed at 6 months of age. Compared with infants with an easy temperament (ET), the Surgency/Extraversion score was significantly reduced in infants with Slow-to-Warm-up temperament (SW). Compared with infants with ET, a trend towards increased Negative Affectivity **(C)** and decreased Orienting/Regulation **(D)**, was observed in the SW temperament infants, although the differences did not reach statistical significance. Data represented as mean ± SD. ∗∗∗ denotes *p* = 0.0008 by unpaired t-test.

This study aimed at defining the effect of maternal exposure to Superstorm Sandy on the placental transcriptome and defining its association with temperament of the offspring in very early life. The differential analysis of the placental transcriptome was conducted retrospectively, by identifying differences between placental samples from children with distinctive temperament profiles (Figure 1A). The description and demographics of the mothers of all participants is provided in Table 1.

### Characteristics of the Infant Temperament and Their Association With Superstorm Sandy-Related Stress

To best evaluate the infant temperament as a whole, we first composited the three temperament dimensions score extracted by the principal component analysis into a standardized z-scores. Based on the z-score, we then divided the infants into three tertiles comprising of equal numbers of children and focused our analysis on the top and bottom tertiles to test our hypothesis. The middle tertile was removed from the analysis to help further reduce variation given our limited sample size. After evaluating the three temperament dimension scores of the top and bottom tertiles, we revealed that, in general, the top tertile with higher factor z-score is characterized as an “Easy Temperament” (ET) profile, whereas the bottom tertile group with lower factor z-score presents characteristics of a “Slow-to-Warm-up” (SW) profile. Typically, infants with Slow-to-Warm-up temperament tend to exhibit a low activity level, are hesitant to engage in, or may withdraw from new situations or stimuli, take their time to adapt to new environments, people and situations, and display a slightly negative mood (Thomas et al., 1970). Specifically, children within the Slow-to-Warm-up group here showed a significantly lower Surgency/Extraversion score compared to the ones within ET group (4.813 vs 5.774, *p* = 0.0008, Figure 1B). No significant difference was detected on Negative Affectivity (3.807 in SW vs. 3.305 in ET, *p* = 0.1751, Figure 1C), while Orienting/Regulation was trending lower in the Slow-to-Warm-up group (4.892 in SW vs. 5.411 in ET, *p* = 0.0883, Figure 1D). There were no significant differences between the Slow-to-Warm-up and Easy Temperament groups in maternal marital status, socioeconomic status, or race (all *p*s > 0.084). A complete summary of the temperament score is shown in Table 1.

We then assessed the relations between temperament variables and Superstorm Sandy maternal stress and maternal psychopathology measures. Correlations among variables and stress factors are shown in Table 2. Only measures comprising the storm stress factor were found to be significantly associated with temperament score (e.g., Surgency/Extraversion), whereas the maternal psychopathological measures were only weakly associated with child temperament.

Next, we performed a hierarchical linear regression in order to estimate the unique contribution of maternal psychopathology and storm stress as predictors of infant temperament. Table 3 shows that storm stress made a unique contribution explaining an additional 18% of the variance in temperament over and above that explained by confounders (step one) and maternal psychopathology (step two). These data suggest maternal prenatal storm stress significantly and uniquely explained infant temperament score beyond what confounders and maternal psychopathology during pregnancy had already explained.

**TABLE 3:** Prenatal maternal stress and psychopathology predict infant temperament.

	Step 1	Step 2	Step 3
Marital status	0.26 (−0.27, 1.28)	0.24 (−0.30, 1.19)	0.26 (−0.21, 1.20)
Race	−0.24 (−0.60, 0.14)	−0.26 (−0.60, 0.11)	−0.30 (−0.62, 0.06)
Socioeconomic status (SES)	−0.01 (−0.69, 0.14)	−0.13 (−0.91, 0.46)	−0.11 (−0.83, 0.46)
Psychopathology factor score	–	0.34 (−0.03, 0.63)	0.38* (0.02, 0.64)
Storm stress factor score	–	–	−0.35* (−0.94, −0.12)
*R* ^*2*^	−0.01	0.07	0.18
*R* ^*2*^ change	0.09	0.11	0.12∗

∗*p* < 0.05. Values represent standardized regression coefficients. Values in parentheses represent 95% confidence intervals.

### Transcriptomic Profile of Placental Tissues Revealed Pathways Enriched in Children With Slow-To-Warm-Up Temperament

**FIGURE 2:**
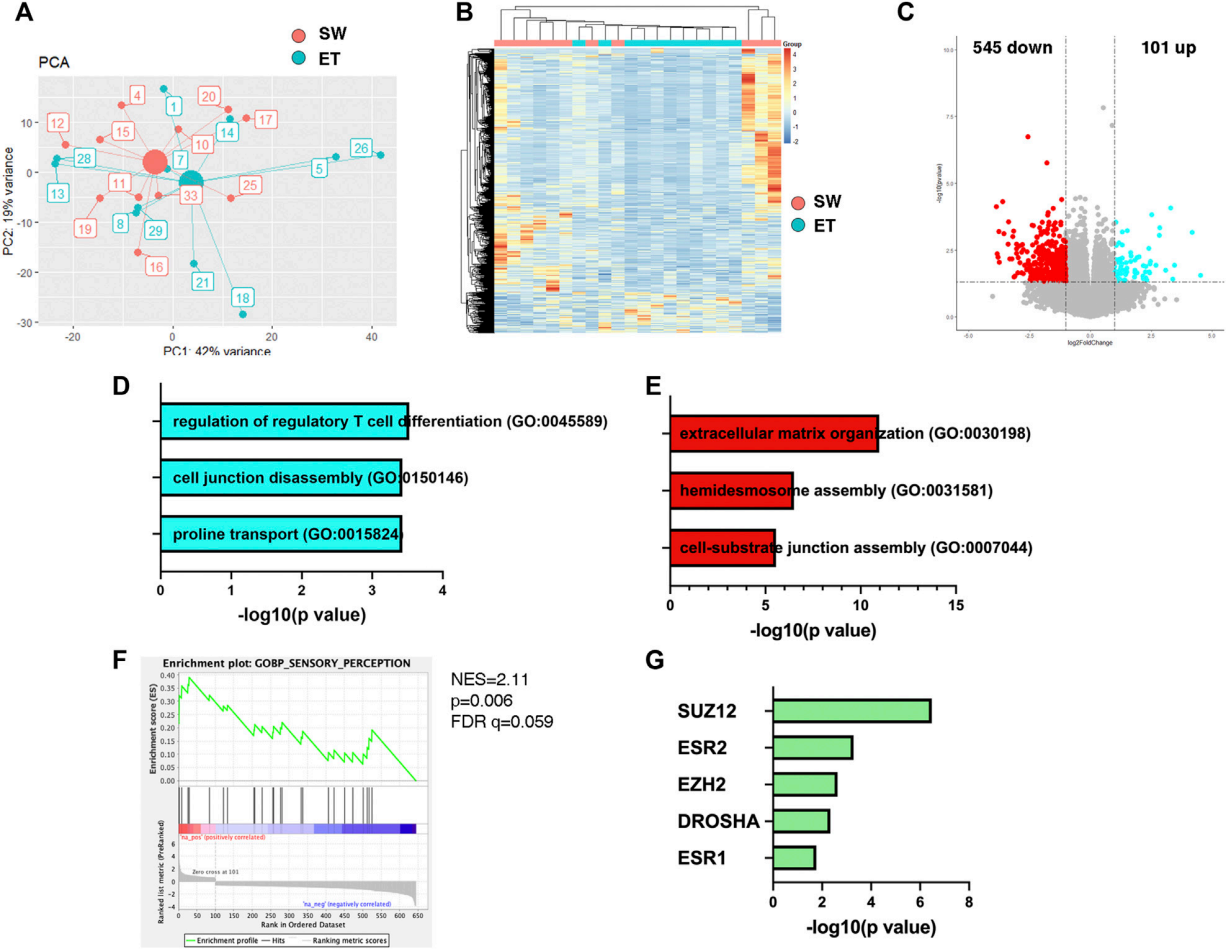
Robust placental transcriptome changes are associated with infant temperament. **(A)** Principal component analysis on the transcriptome of infants with Easy (ET) and Slow-to-Warm-up (SW) Temeprament. Small circles denote each subject, and big circiles represent the centroid of each group. **(B)** Heatmap of placental transcriptome from infants with Easy (ET) or Slow-to-Warm-up (SW) temperament. **(C)** Top differentially expressed genes (*p* < 0.05, fold change >1.5) were identified, with 545 genes downregulated (red) and 101 genes upregulated (blue) in placenta of infants SW temperament. **(D)** The gene ontology category of transcripts with higher expression in the samples from infants with SW temperament, was associated with regulation of regulatory T cell differentiation. **(E)** For the downregulated transcripts in the placental samples form infants with SW temperament, the most significant gene ontology categories related to extracellular matrix organization and cell-substrate interaction. **(F)** In particular, gene set enrichment analysis (GSEA) identified gene sets regulating sensory perception were enriched in the infants with SW temperament. **(G)** ChIP Enrichment Analysis (ChEA) revealed chromatin remodeler in the Polycomb complex 2 (e.g., SUZ12, EZH2), microRNA processing (e.g., DROSHA), and transcription factors in Estrogen Receptor family (e.g. ESR2, ESR1) share the most significant overlapping targets and binding site proximity to the differentially expresses genes in the placental samples from infants with SW temperament.

To define the potential molecular mechanisms by which the maternal environment could influence the mental health of the offspring through placenta, we sought to retrospectively interrogate the differential transcriptomic of placental specimens from infants who were exposed to Superstorm Sandy *in utero* but were characterized with distinct temperament profiles after birth based on the z-score. This post-analysis revealed distinct clustering of the datasets from the two groups of infants with distinct temperament factor scores (Figures 2A,B). Upon differential expression analysis, 646 transcripts were found to be differentially expressed between the two groups (*p < 0.05*, fold change>1.5, Supplementary Table S1). Of those, the levels of 101 transcripts were higher and 545 transcripts were lower in the placental samples from infants with Slow-to-Warm-up Temperament than those from infants with Easy Temperament (Figure 2C, Supplementary Table S1). The transcripts with higher levels in the Slow-to-Warm-up group were enriched for gene ontology categories related to regulation of regulatory T cell differentiation (*CR1*;*TNFRSF18*;*IL2RA*), cell junction disassembly (*DKK1*; *CX3CL1*) and proline transport (*SLC6A15*;*SLC6A20*) (Figure 2D, Supplementary Table S2). The transcripts with lower levels in the Slow-to-Warm-up group were enriched for gene ontology categories related to extracellular matrix organization (*VIT; COL17A1; LAMA3*) and cell-substrate junction assembly (*LAMA3; KRT14; FN1; LAMC2; KRT5; RHOD*) (Figure 2E, Supplementary Table S3). To further explore pathways enriched in the two different temperament profiles, we then performed Gene Sets Enrichment Analysis (GSEA) and focused on gene sets that were enriched in the children with Slow-to-Warm-up Temperament, as they may identify potential pathways relating to environmental exposure, placental transcriptome and child neurodevelopment during early infancy. This analysis of the placental transcriptome identified pathways related to inflammation (e.g. cytokine-cytokine receptor interaction, data not shown) and sensory perception (Figure 2F), which we interpreted as suggestive of links between maternal stress and infant temperament.

We further mined the dataset to start investigating potential molecular mechanisms of regulation of gene expression in the placenta. This was performed by conducting a ChEA analysis of the transcriptome (Supplementary Table S4). This analysis detects putative transcription factors and epigenetic modifiers by comparing the list of differentially expressed genes with published chromatin immunoprecipitation studies (Lachmann et al., 2010). This analysis revealed several chromatin regulators in the Polycomb Repressive Complex 2 (PRC2) family (e.g. SUZ12, EZH2) as well as transcription factors in the Estrogen Receptors family (ESR2, ESR1). Since PRC2 complexes are known modulators of gene repression and most of the differentially expressed genes were down-regulated, the results are of great interest, although further studies are required to validate these data. Finally, DROSHA target genes, were also found to share a large overlap with the differentially expressed genes in the placental transcriptome from the Slow-to-Warm-up group (Figure 2G). Since DROSHA is responsible for microRNA biogenesis, these results collectively suggest that exposure to environmental stressors induces multiple modalities of down-regulation of gene expression.

## Discussion

Adverse maternal experiences during pregnancy have been associated with suboptimal offspring neurodevelopment. Studies of stressful events that impact a large population, such as natural and manmade disasters, offer the advantage of a quasi-experimental approach. In this way, both objective and subjective aspects of stress can be assessed in a population that experienced the same event irrespective of genetic makeup or demographic characteristics. Using unbiased transcriptome analysis and neurobehavioral assessment, we identified a potential link among maternal exposure to Superstorm Sandy, placental gene expression and infant temperament. The link between children’s temperament score and maternal stress exposure was significant. In addition, transcripts related to inflammation and sensory perception were upregulated in the placental samples from children with characteristics of a “Slow-to-Warm-up” temperamental style.

Serving as the interface between the maternal environment and the fetus, the placenta functions as the mediator of stress effects on very early neurodevelopment in their offspring. However, the precise mechanism by which placenta influences offspring’s neurodevelopment is not fully understood. Our study provided several insights on this topic.

First, we identified processes related to T cell differentiation and cytokine-cytokine receptor interaction being upregulated in children with “Slow-to-Warm-up” temperament, suggesting an overall increase in inflammatory response in the placental samples of the group exposed to Superstorm sandy. This is consistent with previous studies reporting placental inflammation in response to prenatal stress and having an effect on offspring’s neurobehavioral phenotype (Davis et al., 2007; Bronson and Bale, 2014; Nomura et al., 2021). It is tempting to speculate that collectively this may suggest a potential role of anti-inflammatory agents to ameliorate placental effects on offspring stress-dysregulation phenotype (Bronson and Bale, 2014).

Second, we detected increased levels of transcripts related to cell junction disassembly and decreased levels of transcripts related to cell-cell junction and extracellular matrix interaction in the placental samples from infants with “Slow-to-Warm-up” temperament profile. Overall this may suggest that environmental stressors may also impact the permeability of the placental epithelium. Since the placenta is a key organ for maternal-fetal communication, these data suggest a dysregulation of the exchange process, which could lead to impaired exchange of metabolites and/or other molecules between the mother and the fetus. Peripheral blood metabolites have been shown to have the ability to reach brain parenchyma and impact neuronal function in the adult brain (Ntranos et al., 2021). This process is even more prominent *in utero*, when the blood brain barrier is not yet formed and maternal and placental metabolites have the ability to flow to the fetus. Further investigation of metabolic changes occurring in the umbilical cord blood from infants from our study cohort will be worth pursuing and provide critical insight on the effect of maternal stress on offspring mental health and neurobehavioral well-being through placenta.

Third, we showed here that exposure to environmental stressors may induce altered placental transcriptome with a predominant effect on down-regulating gene expression, although the underlying molecular mechanisms remain to be further investigated. One proposed mechanism underlying maternal stress-induced long-term adaptions is alteration of the placental epigenome (Conradt et al., 2018; Braun et al., 2020). For example, prenatal maternal depressive symptoms have been associated with lower levels of DNA methylation of *NR3C2* promoter region in placenta tissue (Galbally et al., 2020). A factor score analysis yielded a sex-specific effect on DNA methylation with prenatal stress, in which the *NR3C1* and *HSD11B2* methylation factor scores were associated with hyperactivity and emotion symptoms only in boys (Liu et al., 2021). While our study did not address placental epigenome, we have identified several epigenetic regulators, such as key components of the Polycomb Repressive Complex 2 (PRC2) as well as molecules involved in microRNA processing, to be potential upstream regulators and mediators of the altered placental transcriptome. The PRC2 complex is a well-known regulator mediating transcriptional silencing over large scale gene networks including differentiation, maintaining cell identity and embryonic brain development (Margueron and Reinberg, 2011). MicroRNAs are also known to mediate transcriptional silencing and have been recently implicated as important mediators of early life stress vulnerability to subsequent onset of psychiatric illnesses in adulthood (Allen and Dwivedi, 2020). These findings suggest that exposure to environmental stressors may induce multiple modalities of transcriptional silencing of the placental transcriptome which may subsequently impact early brain development and temperament.

Although only a small subset of differentially expressed genes were upregulated in the placental transcriptome of children with “Slow-to-Warm-up” temperament, we identified genes regulating Sensory Perception as an enriched ontology term in this group. Sensory over-responsiveness has been shown to be more stably correlated with children who were born prematurely (Case-Smith et al., 1998) or who were more fearful and less easily soothed (Van Hulle et al., 2015). Increased sensory features was associated with increased withdrawal and more negative mood in temperament characterization of children with autism spectrum disorder (Brock et al., 2012). Heightened sensory perception has also been frequently reported in children with a “Slow-to-Warm-up” temperament profile (Thomasgard, 2003). Therefore, the identification of enrichment of genes regulating sensory perception is an interesting association of the placental transcriptome with infant temperament. However, these results should be cautiously interpreted with respect to their relevance to brain development.

In conclusion, while the current study bears limitations including small sample size, lack of adequate sex variation and consideration of trimester-specific impact on gene expression and child development, our results suggest a positive association of placental transcriptional network with child early life temperament in response to PNMS. Little is known about the long-term outcomes of infants with “Slow-to-Warm-up” temperament style. Some evidence suggests that Slow-to-Warm-up infants show greater shyness in toddlerhood, which is no longer seen by first grade; however, parenting sensitivity may moderate these developmental trajectories (Grady et al., 2012). Understanding the long-term outcomes for these children is an important topic for future research. Such genetic analysis may provide a powerful tool for assessing risk factors associated with stress-related psychopathological development in the offspring and possibility of developing strategies for early intervention.

## Data Availability

The datasets presented in this study can be found in online repositories. The names of the repository/repositories and accession number(s) can be found in the article/Supplementary Material.
